# Reduced Hippocampal GABA+ Is Associated With Poorer Episodic Memory in Healthy Older Women: A Pilot Study

**DOI:** 10.3389/fnbeh.2021.695416

**Published:** 2021-08-26

**Authors:** Joan Jiménez-Balado, Alexandra Ycaza Herrera, Kay Igwe, Lynda Klem, Korhan Buyukturkoglu, Andrei Irimia, Liu Chen, Jia Guo, Adam M. Brickman, Teal S. Eich

**Affiliations:** ^1^Leonard Davis School of Gerontology, University of Southern California, Los Angeles, CA, United States; ^2^Department of Neurology, Taub Institute for Research on Alzheimer’s Disease and the Aging Brain, Vagelos College of Physicians and Surgeons, Columbia University, New York, NY, United States; ^3^Department of Neurology, Columbia University Irving Medical Center, New York, NY, United States; ^4^Department of Psychiatry, Columbia University, New York, NY, United States; ^5^Corwin D. Denney Research Center, Department of Biomedical Engineering, Andrew Viterbi School of Engineering, University of Southern California, Los Angeles, CA, United States; ^6^Department of Electrical Engineering, Columbia University, New York, NY, United States; ^7^Gertrude H. Sergievsky Center, Vagelos College of Physicians and Surgeons, Columbia University, New York, NY, United States

**Keywords:** episodic memory, γ-aminobutyric acid, GABA, Alzheimer’s disease, sex, apolipoprotein ε4

## Abstract

**Background**: The current pilot study was designed to examine the association between hippocampal γ-aminobutyric acid (GABA) concentration and episodic memory in older individuals, as well as the impact of two major risk factors for Alzheimer’s disease (AD)—female sex and Apolipoprotein ε4 (*ApoE* ε4) genotype—on this relationship.

**Methods**: Twenty healthy, community-dwelling individuals aged 50–71 (11 women) took part in the study. Episodic memory was evaluated using a Directed Forgetting task, and GABA+ was measured in the right hippocampus using a Mescher-Garwood point-resolved magnetic resonance spectroscopy (MRS) sequence. Multiple linear regression models were used to quantify the relationship between episodic memory, GABA+, *ApoE ɛ4*, and sex, controlling for age and education.

**Results**: While GABA+ did not interact with* ApoE* ɛ4 carrier status to influence episodic memory (*p* = 0.757), the relationship between GABA+ and episodic memory was moderated by sex: lower GABA+ predicted worse memory in women such that, for each standard deviation decrease in GABA+ concentration, memory scores were reduced by 11% (*p* = 0.001).

**Conclusions**: This pilot study suggests that sex, but not *ApoE* ɛ4 genotype, moderates the relationship between hippocampal GABA+ and episodic memory, such that women with lower GABA+ concentration show worse memory performance. These findings, which must be interpreted with caution given the small sample size, may serve as a starting point for larger studies using multimodal neuroimaging to understand the contributions of GABA metabolism to age-related memory decline.

## Introduction

Alzheimer’s disease (AD) is a progressive neurodegenerative disorder whose hallmark cognitive symptom is episodic memory loss (Tierney et al., 1996). AD is the leading cause of dementia in the elderly, and disproportionately affects women (Miech et al., 2002; Bacigalupo et al., 2018; Dubal, 2020). Despite decades of research investigating β amyloid (Aβ) as the trigger for a cascade of neuropathophysiological events that cause AD dementia (Hardy and Higgins, 1992), the failure of several high-profile late-stage clinical trials targeting Aβ clearance has highlighted the urgent need to explore alternative causal mechanisms for some key aspects of AD pathophysiology (Cummings et al., 2020). While the cholinergic and glutamatergic systems are known to be affected in AD (Hampel et al., 2018; Findley et al., 2019), the gamma-aminobutyric acidergic (GABAergic) system has received less attention (Pike and Cotman, 1993). However, animal models have shown that GABA plays a critical role in long-term memory formation by synchronizing pyramidal neuron activity (Paulsen and Moser, 1998; Lucas and Clem, 2018) and by preventing hyperactivity in the hippocampus (Najm et al., 2019), a brain structure critical for episodic memory formation and retrieval (Nyberg et al., 1996; Schacter et al., 1996). A study by Li et al. (2021) recently showed, using a 5XFAD AD-mouse model, that hyperactivity of pyramidal neurons in the CA1 field of the hippocampus was driven by GABA_A_ receptor-mediated inhibitory synaptic decline, preceded Aβ-related pathology, was accompanied by cognitive impairments in an episodic-like memory task, and could be reversed *via* administration of a GABA_A_ receptor agonist (Li et al., 2021). In humans, electrophysiological hyperactivity in the hippocampus—a brain structure that undergoes early and significant morphologic changes in AD (Putcha et al., 2011)—presages episodic memory decline in individuals at-risk for AD (Dickerson et al., 2005; Hämäläinen et al., 2007; Sperling et al., 2010; Yassa et al., 2010). Levetiracetam (Keppra), an anti-epileptic drug thought to enhance the function of GABA indirectly and to target hyperexcitability, reduces hippocampal hyperactivity, as indicated by decreased blood oxygenation level-dependent (BOLD) activation measured *via* functional magnetic resonance imaging (fMRI). Levetiracetam also mitigates memory impairment in patients with amnestic mild cognitive impairment (Bakker et al., 2012, 2015). Together, these findings suggest that GABAergic dysfunction plays a key role in the early hippocampal hyperactivity that is associated with episodic memory impairments in people at risk for, and with, AD.

The prevalence of AD is greater in women than in men (Miech et al., 2002; Bacigalupo et al., 2018; Dubal, 2020). This higher rate may reflect the fact that women typically live longer than men (Mielke, 2018), and/or a sex dimorphism involving either organizational effects that occur during development (Carroll et al., 2010; Luo et al., 2020) or activation effects occurring in mid-to-late life, most notably in the form of age-related estrogen reductions (Pike, 2017; Dubal, 2020). Estradiol (E2), the primary bioactive estrogen in women, increases spontaneous GABA release and increases the expression of GABA_A_ receptors (Herbison et al., 1990; Herbison and Fénelon, 1995). Along with the decline in E2 levels post-menopause, GABA levels (at least in the anterior cingulate cortex) have been reported as significantly lower than pre-menopausal ones (Wang et al., 2019). Pathology studies in humans have shown lower expression of GABA_A_ α1, α2, α5, β3 receptor subunits on the membranes of brain neurons in healthy older females in regions like the superior temporal gyrus (Pandya et al., 2019). Furthermore, *in vivo* studies of the frontal cortex suggest that there are stronger negative correlations between GABA levels and age in women than in men (Gao et al., 2013).

The Apolipoprotein ɛ4 allele (ApoE ε4) is the strongest common genetic risk factor for late-onset AD, being associated with both higher risk and a markedly earlier mean age of AD onset (Corder et al., 1993; Cacabelos, 2003). Several studies comparing cognitively-normal ɛ4 carriers to non-carriers reported memory-related electrophysiological hyperactivity in the hippocampus and entorhinal cortex (Bondi et al., 2005; Dickerson et al., 2005; Filippini et al., 2009; Sperling et al., 2010). *In vivo* animal studies have shown that learning and memory losses can be rescued through the deletion of ApoE ε4 in GABAergic interneurons (Knoferle et al., 2014) and that GABA-expressing interneurons in the hippocampus are selectively vulnerable to ApoE ɛ4-mediated neurotoxicity, including decreases in dendritic arborization and spine density (Jain et al., 2013). Indeed, Najm et al. (2019) recently proposed that GABAergic interneurons are selectively vulnerable to *ApoE ɛ4*, which may translate into a reduction of phasic and tonic inhibition that results in hippocampal excitability (Najm et al., 2019).

Thus, the relation between dysfunction in hippocampal GABA signaling and age-related memory impairment has been widely studied using animal models (Ambrad Giovannetti and Fuhrmann, 2019; Najm et al., 2019), and human studies have revealed interactions between hippocampal hyperactivity and memory which may serve as a biomarker for impending AD. Nevertheless, to our knowledge, no study to date has tested whether hippocampal GABA is associated with episodic memory in cognitively healthy older adults, or considered how such a relationship may be moderated by AD risk factors including sex or ApoE ε4 genotype. The current pilot study explores whether ApoE ε4 and/or sex are associated with decreases in hippocampal GABA concentration and, if so, whether such decreases predict worse episodic memory performance. Briefly, participants completed an episodic memory task, and a Mescher-Garwood point-resolved spectroscopy sequence (MEGA-PRESS) was then used to measure GABA concentration in the right hippocampus, allowing us to interrogate the effects of GABA concentration, ApoE ε4, and sex, as well as their interactions, upon episodic memory.

## Materials and Methods

### Setting and Participants

Healthy older adults were recruited for the study from two participant cohorts maintained by the Cognitive Neuroscience Division at Columbia University, the Cognitive Reserve Study, and the Reference Ability Neural Network Study. Participants were recruited to these studies by mail-market procedures targeting individuals within 10 miles of the Columbia University Medical Center. Participants were required to be right-handed, native English speakers with at least a fourth-grade reading level. As part of these cohort studies, participants were genotyped for ApoE ε4 and screened for neurological diagnoses and medication use, as detailed elsewhere (Stern et al., 2014), and for dementia using the Dementia Rating Scale (Mattis, 1988). Any participant who scored below 135 was excluded. From this cohort pool, we recruited participants based on their ɛ4 carrier status (ɛ4+ and ɛ4-) and sex (male and female), to obtain a final sample balanced across both variables. Participants performed a Directed Forgetting memory task (MacLeod, 2012) and then underwent MRI scans at the New York State Psychiatric Institute MRI Research Program. Data from 11 women and nine men aged 50–71 years (y) were included. The median age of the sample was 61 years (y; range: 54.5 y to 67.8 y). Ten women self-reported to be postmenopausal. Data on the 11th woman were not available. Twelve participants were ApoE ε4^+^ (ε2/ɛ4 = 1; ε3/ɛ4 = 10; ɛ4/ɛ4 = 1), and eight were ApoE ε4^−^ (ε3/ε3 = 7; ε2/ε3 = 1). The median education level was 6 (range: 5–7), which corresponds to a bachelor’s degree or equivalent, according to the International Standard Classification of Education (ISCE) classification. Written informed consent, as approved by the Institutional Review Board of the Columbia University Medical Center, was obtained prior to study participation.

### Directed Forgetting Task

An item-method directed forgetting task was used to assess episodic memory (MacLeod, 2012). In the study phase of the task, participants were presented with unrelated, unambiguous concrete nouns, ranging in length from 3 to 8 letters, one at a time, for 2,500 ms each. Each word was followed by a 500 ms delay, and then a memory cue, presented for 1,500 ms, which indicated whether the preceding word was to be remembered (TBR) or to be forgotten (TBF) for a later memory test. Participants were instructed to remember the TBR words for a later memory test and told that forgetting the TBF words would help them to remember all of the TBR words. The TBR cue consisted of four green R’s (for Remember), and the TBF cue consisted of four red F’s (for Forget). To minimize primacy and recency effects, six additional buffer trials were presented as the first and last three trials of the experiment and were not scored. Trials were separated by 1,000 ms intervals. Following the study phase, and after a 5-min delay period, memory was tested for all 36 studied words (18 TBR and 18 TBF), as well as 36 words that had not been presented during the study phase. Old and new words were presented in a blocked-randomized design to control for the time between study and test. During this recognition phase, each test word was presented on the screen for 20 s, or until the participant responded. Participants were instructed to press the Y key on the keyboard (for Yes) if they recognized the test word as one of the words that had been presented to them, and to press the N key (for No) if it had not. The current analysis examined only accuracy for TBR items.

### Neuroimaging Protocol

#### Magnetic Resonance Imaging

**Figure 1:**
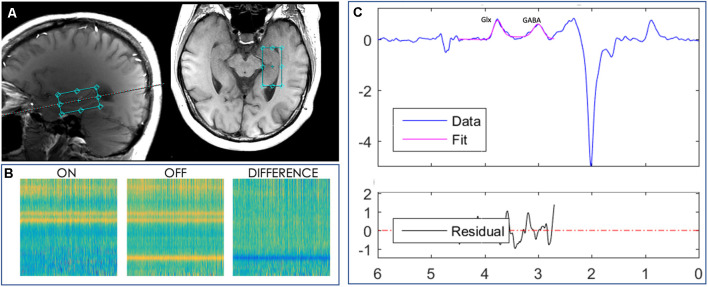
Localized images and representative MR spectra from a 2 × 2 × 4 cm^3^ voxel manually placed in the right hippocampus of a subject. **(A)** Axial and sagittal planes showing the hippocampal voxel, outlined in aqua, from one study participant’s MPRAGE T1-weighted image. **(B)** Loadings for the GABA edited difference spectrum. **(C)** Representative model fitting showing Glx (a combination of glutamate and glutamine) and GABA spectrum peaks, representing the GABA signal, from the same subject. The blue line represents the actual edited spectrum, whereas the overlaid pink line is the model of best fit. The residual is shown in the black curve below the modeling plot.

MRI data were acquired using a 32-channel head coil on a 3 Tesla MR scanner (Discovery, GE Medical Systems). Two anatomical images were acquired for the MRS volume of interest (VOI) placement; the first one was a three-dimensional (3D) brain volume (BRAVO) *T*_1_-weighted sequence (echo time (*T*_E_) = 2,700 ms, repetition time (*T*_R_) = 7,156 ms, inversion time (*T*_I_) = 450 ms, 176 slices, 256 × 256 matrix size, slice thickness = 1 mm, flip angle (FA) = 12°). The second one was a two-dimensional (2D) axially-acquired structural *T*_1_-weighted fluid-attenuated inversion recovery (FLAIR) volume (*T*_E_ = 26 ms, *T*_R_ = 2,300 ms, *T*_I_ = 756 ms, 25 slices, 512 × 512 matrix size, slice thickness = 5 mm, voxel size = 0.4 mm × 0.4 mm × 5 mm, FA = 111°). The VOI with a size of 4 × 2 × 2 cm^3^ was centered in the right hippocampus (Figure 1A). ^1^H MRS data were acquired using a MEGA-PRESS sequence (Mullins et al., 2014; *T*_E_ = 68 ms, *T*_R_ = 1,500 ms, slice thickness = 20 mm, FA = 90°, field of view = 512 × 512) in one acquisition that lasted 768 s. A vendor-provided, semi-automatic shimming procedure was implemented prior to spectroscopic acquisition and was supplemented by interactive manual shimming, resulting in full-width at half-maximum (FWHM) water linewidths ranging from 9 to 22 Hz (mean line width = 13 ± 3.49 Hz).

#### Anatomical Segmentation

The 3D T1-weighted images were analyzed using FreeSurfer (v5.1.0) an automated segmentation and cortical parcellation software package (Fischl et al., 2002). Boundary lines separating gray matter, white matter, and pial surfaces were visually inspected. When necessary, to ensure accuracy, manual editing of voxel label maps was conducted according to the FreeSurfer manual editing guidelines by a technician blinded to participant demographics. In the second round of quality control, the borders of the parcellated cortical and sub-cortical regions were then overlayed onto the input structural images by a second technician. The Desikan-Killiany Atlas, which includes 34 gyral-based cortical regions, was used for cortical parcellation and for regional identification of clusters (Desikan et al., 2006).

#### Magnetic Resonance Spectroscopy Quantification

The concentration of resting-state GABA in the right hippocampus was quantified using the Jet algorithm^1^ in MATLAB (Mathworks, MA, USA). This algorithm was used to preprocess the spectroscopy data by aligning frequency and phase for ON and OFF spectra, as described previously (Mikkelsen et al., 2018). Then, we edited the GABA peak at 3 parts per million (ppm) and co-edited the glutamine + glutamate (Glx) peak at 3.77 ppm after subtraction of the ON and OFF spectra, as shown in Figure 1B. Spectral fitting was performed with a simulated basis-set for metabolites including GABA, Glx, choline (Cho), creatine (Cr), and n-acetylaspartate (NAA). Metabolites were quantified based on the area-under-the-curve (AUC) for each fitted metabolite basis, as illustrated in Figure 1C. The signal detected with these parameters will contain contributions from both the macromolecules (MM) and homocarnosine in addition to GABA (Rothman et al., 1997), therefore we refer to this signal as GABA+ henceforth. GABA+ concentration was then quantified as a ratio to the reference Cr metabolite concentration.

#### Statistics

The proportion of correct TBR responses (items that participants were told to remember, which they correctly said were presented), which provides a direct measure of episodic memory, served as the outcome variable. Predictors of interest included MRS-measured GABA+ concentration from the right hippocampus, age, sex, education level (according to the International ISCE classification), and ApoE ε4 genotype, coded as a binary variable (either positive or negative). There were no missing data pertaining to any of these variables.

Univariate analyses were used to assess the association between GABA+, on the one hand, and age, sex, and ApoE ε4 variables, on the other hand, using Spearman’s rank correlation coefficients or Student’s *t-*tests for continuous or categorical variables, respectively. Multiple linear regression models were used to evaluate the relationship between episodic memory and the predictors of interest. Memory served as the dependent variable, with GABA+, age, sex, education, and ApoE ε4 as independent variables. Independent variables were selected according to *a priori* hypotheses based on the previous literature or on univariate analysis results. To facilitate the interpretation of regression coefficients, GABA concentrations were standardized into *z*-scores. As our hypothesis involved ApoE ε4 and sex effects on GABA+ concentrations, interaction terms for ApoE ε4 × GABA+ and sex × GABA were included. Additionally, to avoid overfitting due to the large number of variables and small sample size, variables were selected *via* backward stepwise elimination according to the Akaike information criterion (AIC). Briefly, the AIC is a metric comparing the goodness of the fit of two models by selecting the one with the highest likelihood after penalizing for the number of parameters in the models. A lower AIC thus corresponds to better goodness of fit. The statistical assumptions (independence and normality of residuals, presence of influential cases, and absence of multicollinearity) of the model obtained through variable selection were verified to confirm that they had been met.

Overall accuracy on the task was high. To investigate as to whether the effect of GABA+ (the predictor variable) on memory was conditioned by the skewed distribution of TBR responses (the dependent variable), we implemented separate quantile regression models in men and women. In quantile regression, instead of fitting a model at the mean of the dependent variable, the effect of the independent variable is tested across the distribution of the dependent variable. Hence, coefficients are calculated at one or more quantiles of the distribution (expressed as τ), which are set *a priori*. In our case, we considered deciles from 10 to 90. This analysis allowed us to observe whether the correlation between GABA+ and memory remained constant across the distribution of TBR responses in men and women, giving robustness to our results. Pairwise comparisons of those models fitted at different τ were compared using Wald tests to assess whether the effect of GABA+ varied across the distribution of TBR responses.

All analyses were conducted using R software (R version 3.6.1, 2019-07-05; © 2019 The R Foundation for Statistical Computing). For all analyses, α was set at 0.05.

### Results

#### Characteristics of the Sample

There was no significant sex-related difference in the sample’s age (*U*_(9,11)_ = 56.5, *p* = 0.621), educational attainment (*U*_(9,11)_ = 62.5, *p* = 0.318), or in its prevalence of the ApoE ε4 allele ($\chi_{20}^{2}$ = 0.01, *p* = 0.927). On the other hand, ApoE ε4 carriers were older [median (interquartile range) = 65.5 y (55.8, 69.0) y] than non-carriers [57.5 y (53.8, 60.3) y], but did not differ in educational attainment (*U*_(8,12)_ = 64.5, *p* = 0.194).

#### Relation Between GABA Concentration and Episodic Memory

The average proportion of correctly recognized TBR words was high, 0.9 ± 0.1. This score did not correlate with age (*rs*_18_ = 0.32, *p* = 0.169) or education level (*rs*_18_ = 0.11, *p* = 0.632). Further, GABA+ was not associated with either age (*rs*_18_ = 0.05, *p* = 0.828), or *ApoE ɛ4* polymorphism (*t*_(14,5)_ = 0.26, *p* = 0.802). However, overall, women had higher GABA+ concentration than did men (*t*_(16,9)_ = −2.67, *p* = 0.016).

**Figure 2:**
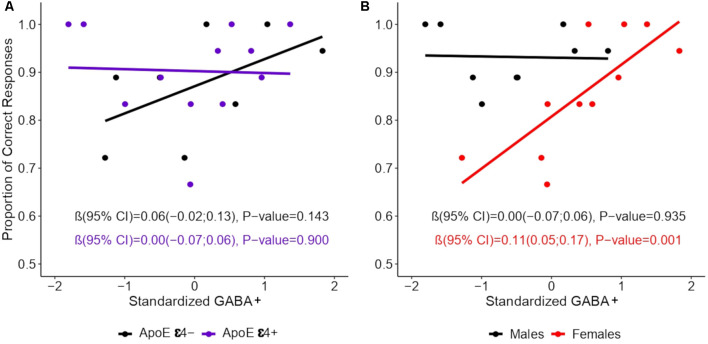
Association between episodic memory and GABA+ concentration by sex. GABA+ concentration was standardized into *z*-scores. Lines show the relation between GABA+ levels and episodic memory performance by **(A)** ApoE ε4 and **(B)** Sex. Linear regression models were constructed using the proportion of the TBR words (those words that participants were instructed to remember) as the dependent variable, and while accounting for the interactions of ApoE ε4 × GABA+ level and of sex × GABA+ level in separate models. Values represent regression coefficients β, their 95% confidence intervals (CIs), and the *p*-values of the standardized GABA main effects.

Multiple linear regression models were used to analyze the relationship between episodic memory and the predictors of interest (age, education, sex, and ApoE ε4 genotype), the results of which are shown in Table 1. We did not observe a main effect of ApoE ε4 [*β* (95% confidence interval) = −0.02 (−0.13, 0.10), *p* = 0.757], or a ApoE ε4 × GABA+ interaction [*β* = 0.00 (−0.11, 0.11), *p* = 0.990; see Figure 2A]. We did observe a main effect of sex, such that, on average, women had worse memory performance [0.81 (0.75, 0.86)] than men [0.93 (0.87, 0.99)]. However, this main effect was moderated by a significant interaction between GABA+ concentration and sex, such that lower GABA+ concentrations were associated with worse memory performance in women (Table 1), but not in men: *β* = 0.00 (−0.07, 0.06), *p* = 0.935. As shown in Figure 2B, in women, for each standard deviation decrease in GABA+ concentration, the proportion of correct responses on the memory task decreased by 0.11.

**Table 1:** Linear regression models parameters illustrating the relationship between GABA+ concentration and episodic memory performance.

Variable	β(95% CI)	*t*-value	*p*-value
	Baseline Model		
Age [years]	0.00 (−0.01;0.01)	0.69	0.501
Education, ISCED	−0.01 (−0.07;0.04)	−0.61	0.551
GABA+ level, SD increase	0.11 (0.04;0.18)	3.21	0.007
*ApoE ɛ4*, Positive	−0.02 (−0.13;0.10)	−0.32	0.757
Sex, Male	0.12 (0.02;0.22)	2.72	0.019
GABA+ × *ApoE ɛ4*	0.00 (−0.11;0.11)	0.01	0.990
GABA+ × Sex	−0.12 (−0.24;0.00)	−2.17	0.051
	**Final model**
GABA+, SD increase	0.11 (0.05;0.17)	3.92	0.001
Sex, Male	0.12 (0.04;0.21)	3.10	0.007
GABA+ × Sex	−0.11 (−0.20;−0.03)	−2.74	0.015

*Linear regression models were constructed using the proportion of correctly recognized TBR words as the dependent variable, while adjusting for GABA+, age, sex, education, the presence of the ApoE ε4 allele, and for both the GABA+ level × ApoE ε4 and GABA+ level × sex interactions. The final model displayed was obtained after selecting variables *via* backward stepwise elimination. Female sex is the reference category; thus, the main effect of GABA+ level showed in the final model represents the association between GABA+ concentration and episodic memory performance in females. The adjusted *R*^2^ values were 0.34 and 0.48 for the baseline and final models, respectively. Listed are β coefficients and their 95% confidence intervals, *t*-statistics, and *p*-values*.

**Figure 3:**
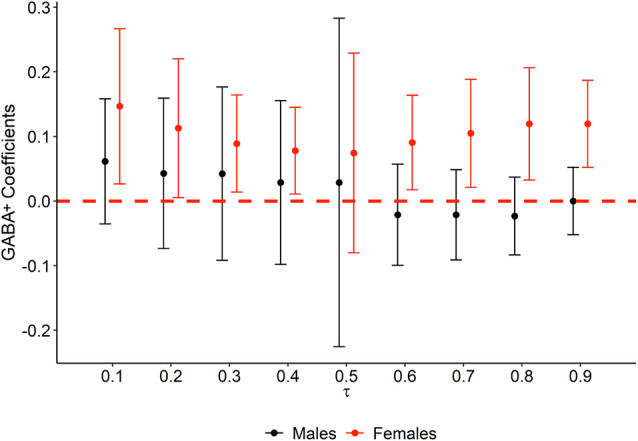
Quantile regression plot of GABA+ statistical effect on memory performance at each decile of TBR accuracy, by sex. The variation of GABA+ regression β coefficients (*y* axis) obtained from quantile regression models is represented at each decile (*x* axis). Colors represent males (black) and females (red). The red dashed line is set at 0, and thus error bars not crossing the red dashed line represent statistically significant associations.

The results of the quantile regression models revealed that GABA+ was not associated with memory performance in men in any portion of the TBR accuracy distribution. By contrast, in women, GABA+ was positively correlated with memory at all deciles except 20 and 50 (*τ*^10^ = 0.10, *t* = 2.39, *p* = 0.040; *τ*^20^ = 0.20, *t* = 2.06, *p* = 0.070; *τ*^30^ = 0.30, *t* = 2.32, *p* = 0.045; *τ*^40^ = 0.40, *t* = 2.27, *p* = 0.049; *τ*^50^ = 0.50, *t* = 0.94, *p* = 0.370; *τ*^60^ = 0.60, *t* = 2.43, *p* = 0.038; *τ*^70^ = 0.70, *t* = 2.46, *p* = 0.036; *τ*^80^ = 0.80, *t* = 2.70, *p* = 0.025; *τ*^90^ = 0.90, *t* = 3.47, *p* = 0.007). As shown in Figure 3, GABA+ related regression coefficients in women ranged from 0.08 to 0.15. When significant models were compared by pairs, no significant differences in any comparison were found, suggesting relative stability of the GABA+ concentration effect in women and confirming that these results were not conditioned by a potential ceiling effect observed in TBR accuracy.

## Discussion

To our knowledge, this is the first study to test the relation between hippocampal GABA+ and episodic memory in older adults. Contrary to our expectations, *ApoE ε4* status did not moderate the effect of GABA+ concentration on memory. However, sex did: women with lower GABA+ concentrations showed worse episodic memory compared to women with higher GABA concentrations and to men, regardless of the latter’s GABA+ concentration. What factors might mediate this effect? The female hippocampus is very responsive to E2. In women, hippocampal volume increases during the high-estradiol late-follicular phase of the menstrual cycle (Protopopescu et al., 2008). In animal models, the dendritic spine density of pyramidal hippocampal neurons increases during the high-estradiol proestrous phase (Woolley et al., 1990), resulting from decreased GABAergic inhibition in the hippocampus (Murphy et al., 1998). The changes in the hippocampal GABA system from pre- to post-menopause—dynamic fluctuations across the menstrual cycle (Protopopescu et al., 2008) to static low levels—may result in static hyperexcitability of hippocampal neurons and to increased risk of pathophysiology.

Other potential overlapping mediating factors are depression and cognitive impairment. While the results are not always consistent, both case-control and cohort studies have reported that a history of depression is a risk factor for cognitive impairment (Kessler, 2003; Ownby et al., 2006), and increases AD risk (Ownby et al., 2006). Women have a higher prevalence of depression (Pehrson and Sanchez, 2015; Flores-Ramos et al., 2017), with symptom risk peaking during major reproductive events (e.g., perimenopausal transition) when fluctuations in sex steroid hormone levels are high (Soares and Zitek, 2008). These transitional phases are associated with dysregulation of the hypothalamic-pituitary-gonadal axis function (Schweizer-Schubert et al., 2021), which is regulated by GABAergic transmission (Flores-Ramos et al., 2017). Interestingly, individuals with major depression have reduced numbers of somatostatin-expressing neurons (a population of GABAergic interneurons playing a key role in memory), and this reduction is exacerbated in women (Fee et al., 2017). Unfortunately, as this was a pilot study, we did not acquire sex hormone levels or screen for depressive symptoms, and we were therefore unable directly to test hypotheses on the role of these factors in the relationships between GABA concentrations, episodic memory, and sex that we quantified in this study.

Recently, Schmitz et al. (2017) reported an association between MRS-measured hippocampal GABA+ and the mnemonic control over unwanted thoughts (Schmitz et al., 2017). However, this study included only younger adults (Mean age = 24.7 year). A study by Porges et al. (2017) did investigate the relation between GABA+ and cognitive decline in older individuals (Porges et al., 2017). However, the neuropsychologic measure used in their study (the Montreal Cognitive Assessment, MoCA) was broad and cognitively non-specific, and GABA concentration was assayed in the prefrontal cortex, not in the hippocampus.

Correlations between GABA+ in other brain regions and other cognitive functions have been reported. Riese et al. (2015), for example, reported better performance in a word list task for older individuals with greater GABA+ concentration in the posterior cingulate cortex (Riese et al., 2015). Likewise, several studies reported that GABA+ concentrations in the dorsal anterior cingulate and in the occipital cortex are associated with measures of executive and visuo-perceptual functions, respectively (Marenco et al., 2018; Simmonite et al., 2019). Furthermore, Piras et al. (2019, 2020) found cerebral GABA levels to be associated with performance in phonemic fluency and in the Stroop Color-Word Test, a measure of response inhibition (Piras et al., 2019, 2020). Thus, it is possible that the relation between GABA+ levels and cognition is not specific to memory. However, the data presented here, while drawn from a small sample, support findings from animal models, which have provided strong evidence for the specificity of the age-related, GABA-mediated hippocampal-episodic memory association.

In summary, the data from this pilot study revealed an association between GABA+ levels and episodic memory in women but not men, such that lower levels of GABA+ were associated with worse behavioral performance. Further multimodal neuroimaging studies considering structural, MRS, and fMRI data are needed to determine whether these GABAergic changes are also associated with hippocampal hyperactivity (Jiménez-Balado and Eich, 2021). Moreover, longitudinal studies with larger samples that consider depression and hormonal balances will help to replicate the findings presented here and test whether GABA-related dysregulation predicts sex-specific incident MCI or dementia risk. Further studies focusing on these questions would be of great interest in confirming the contribution of GABA to age–related cognitive impairment, and clarifying the role of sex in these changes.

## Limitations

This pilot study is preliminary and, as such, has several notable limitations that necessitate the results to be interpreted with caution. First, the sample size was small, which limited statistical power, especially for the critical analyses of group comparisons. Second, we collected neither sex hormone levels (estradiol, progesterone, and testosterone) nor current levels or history of depression. These are important avenues of future inquiry, as they may provide insight into the mechanism driving the sex-specific effects found, and future studies should directly test the role of these factors in the relationship between GABA+ concentration and episodic memory in women. Third, while it is not possible to determine from the ^1^H MRS estimate where the GABA signal originates, as the measurement reflects a combination of synaptic, intracellular, and extrasynaptic GABA from all types of GABAergic interneurons in our right hippocampal region of interest (Maddock and Buonocore, 2012), the findings reported by Li et al. (2021) suggest that hippocampal CA1 GABA_A_ postsynaptic pyramidal neuron receptors might be a likely source. Future studies using PET imaging could provide clarity on the precise coupling of the source of the GABA signal and its association with episodic memory deficits. Moreover, fMRI measurements will additionally help to ensure that the effect of GABA reduction or dysfunctional coupling on cognitive impairment is mediated by hippocampal hyperactivity; confirming the main hypothesis of this manuscript. Finally, our sample may not be representative of typical older adults, according to both their self-reported levels of education, and to their objective (high) performance on the memory task. On the other hand, hippocampal volume in our sample (μ ± *σ* = 3.84 ± 0.5 cm^3^) was on par with recently published normative data acquired from a large sample (N ≃ 20,000) of clinically healthy older adults (mean age: 62.95 ± 7.48 y; hippocampal volume ≃3.86 ± 0.4 cm^3^), and these results were similar across sex. Specifically, the women in our sample had average hippocampal volumes of 3.64 ± 0.47 cm^3^, vs. 3.77 ± 0.37 cm^3^ in the normative sample. Men in our sample had average hippocampal volumes of 4.12 ± 0.35 cm^3^, vs. 3.97 ± 0.43 cm^3^ in the normative sample, which allays some concern over how representative the participants in our sample are (Nobis et al., 2019). Future studies that include larger samples, use multimodal imaging methods, and also measure depression and hormone levels would help to both remedy these limitations and to facilitate generalization of the results to the general population.

## Data Availability Statement

Data supporting the conclusions of this manuscript will be shared under petition of qualified researcher.

## Ethics Statement

The studies involving human participants were reviewed and approved by The Institutional Review Board of the Columbia University Medical Center. The patients/participants provided their written informed consent to participate in this study.

## Author Contributions

JJ-B performed statistical analysis, created visualizations, and wrote the manuscript. AY wrote the manuscript. LK acquired behavioral and MRS data. KI and CL processed and analyzed the MRS imaging data. KB set up the MRS sequence and helped acquire the MRS data. JG provided technical support for the MRS analysis software. AI and AB edited the manuscript. TE conceived the study, acquired behavioral and MRS data, analyzed and interpreted data, wrote the manuscript, and acquired funding. All authors contributed to the article and approved the submitted version.

## Conflict of Interest

The authors declare that the research was conducted in the absence of any commercial or financial relationships that could be construed as a potential conflict of interest.

## Publisher’s Note

All claims expressed in this article are solely those of the authors and do not necessarily represent those of their affiliated organizations, or those of the publisher, the editors and the reviewers. Any product that may be evaluated in this article, or claim that may be made by its manufacturer, is not guaranteed or endorsed by the publisher.

## Footnotes

^1^ http://triton.iqfr.csic.es/guide/man/nmrsim/contents.html
